# Application of kernel principal component analysis for optical vector atomic magnetometry

**DOI:** 10.1088/2632-2153/ad0fa4

**Published:** 2023

**Authors:** James A McKelvy, Irina Novikova, Eugeniy E Mikhailov, Mario A Maldonado, Isaac Fan, Yang Li, Ying-Ju Wang, John Kitching, Andrey B Matsko

**Affiliations:** 1 Jet Propulsion Laboratory, California Institute of Technology, Pasadena, CA 91109, United States of America; 2 Physics Department, College of William & Mary, Willaimsburg, VA 23187, United States of America; 3 National Institute of Standards and Technology, Boulder CO 80305, United States of America

**Keywords:** kernel principal component analysis, vector magnetometry, unsupervised machine learning, support vector regression machines

## Abstract

Vector atomic magnetometers that incorporate electromagnetically induced transparency (EIT) allow for precision measurements of magnetic fields that are sensitive to the directionality of the observed field by virtue of fundamental physics. However, a practical methodology of accurately recovering the longitudinal angle of the local field through observations of EIT spectra has not been established. In this work, we address this problem of angle determination with an unsupervised machine learning algorithm utilizing nonlinear dimensionality reduction. The proposed algorithm was developed to interface with spectroscopic measurements from an EIT-based atomic rubidium magnetometer and uses kernel principal component analysis (KPCA) as an unsupervised feature extraction tool. The resulting KPCA features allow each EIT spectrum measurement to be represented by a single coordinate in a new reduced dimensional feature space, thereby streamlining the process of angle determination. A supervised support vector regression (SVR) machine was implemented to model the resulting relationship between the KPCA projections and field direction. If the magnetometer is configured so that the azimuthal angle of the field is defined with a polarization lock, the KPCA-SVR algorithm is capable of predicting the longitudinal angle of the local magnetic field within 1 degree of accuracy and the magnitude of the absolute field with a resolution of 70 nT. The combined scalar and angular sensitivity of this method make the KPCA-enabled EIT magnetometer competitive with conventional vector magnetometry methods.

## 1. Introduction

Emerging methods in machine learning and the continued maturation of embedded system processors has enabled the development of smart sensor technologies within the field of metrology [1–4]. Joint optimization of sensor design and statistical signal processing allows for the efficient extraction of complex relationships within sensor data, resulting in improved sensitivity and speed in sensor performance. Unsupervised machine learning techniques, such as those used for clustering and dimensionality reduction, have allowed for numerical modeling of physical processes without the need for initial assumptions of the underlying physics. In atomic, molecular, and optical (AMO) spectroscopy, machine learning techniques have been used for regression problems such as absorbance measurement [5, 6], signal restoration [7, 8], density estimation [9] and quantum state reconstruction [10]. Furthermore, applications have been found in classification problems for the identification of light sources [11, 12], near infrared spectroscopy [13], and electron microscopy [14].

Optical vector atomic magnetometry is a maturing AMO research area that stands to benefit from these data-driven modeling methods, due to the complexity of the physics involved. Atomic magnetometers are typically scalar sensors that measure transitions between magneto-sensitive spin sublevels of the ground state of alkali atoms. These instruments boast both high accuracy and sensitivity, and have been found to be valuable in underwater anomaly detection as well as planetary science [15–18]. At the time of writing, the highest performing scalar atomic magnetometers utilize spin-exchange relaxation free (SERF) techniques [19, 20], and can achieve sensitivity on the order of $1\;fT/\sqrt{Hz}$. However, SERF magnetometers are limited to scalar measurements at low magnetic fields, and are therefore ill-suited to unshielded, outdoor applications. In addition to the SERF designs, an assortment of other atomic magnetometers have been developed and commercialized [21].

Vector atomic magnetometry is typically performed by taking measurements with properly optimized scalar magnetometers. For instance, introducing low frequency AC fields along a set of coordinate axes that have been defined within a laboratory frame results in vector detection [22]. This approach requires an intricate coil system and fundamentally relies on physically aligning the orthogonality of the laboratory coordinate system. Consequently, any misalignment in the field orientation introduces errors in the determination of the field direction as well as cross-axis sensitivity.

**Figure 1.**
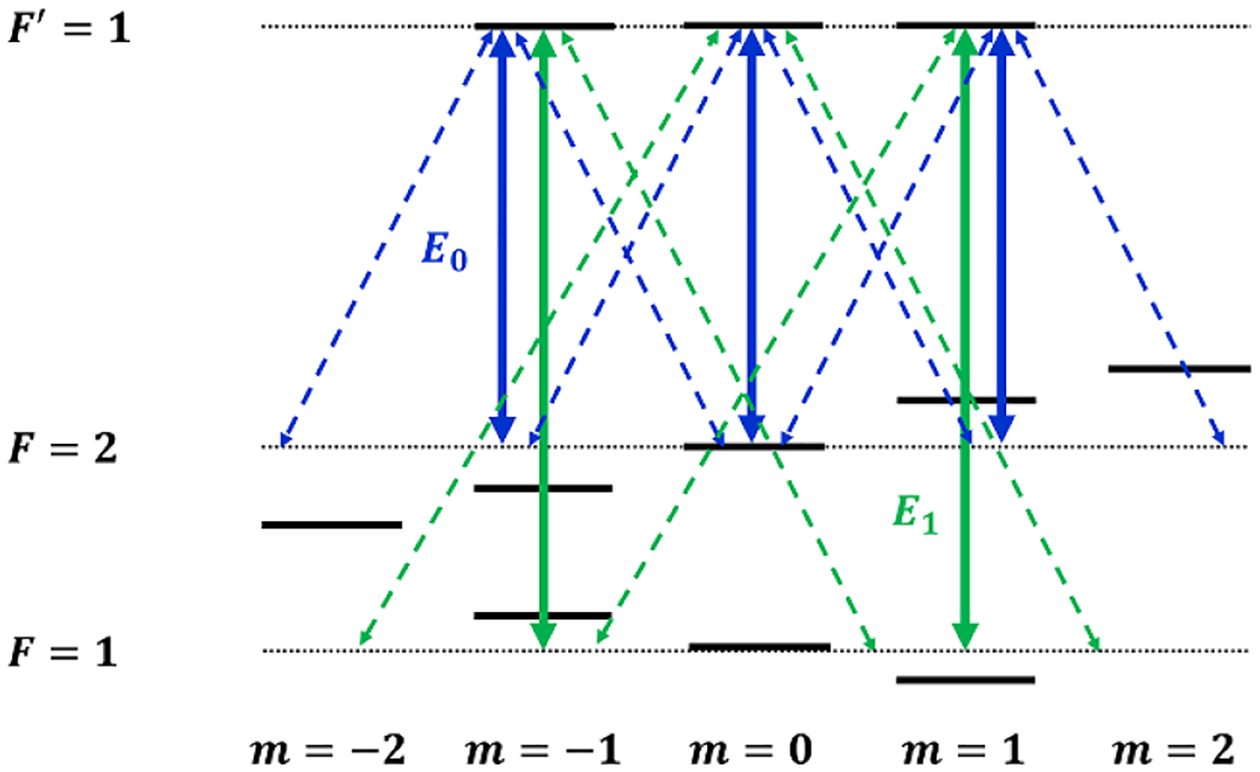
Relevant atomic levels for ^87^Rb $D_{1}\left(5^{2}S_{1/2}\mapsto 5^{2}P_{1/2}\right)$ optical transition. Atoms are prepared with linearly (lin||lin) polarized optical fields $E_{0}$ and $E_{1}$, which have carrier frequencies separated by the ground-state hyperfine splitting (6835 MHz). Arrows indicate possible optical transitions between various Zeeman sublevels of the hyperfine states. The transitions shown by solid (dashed) arrows are realized for the magnetic field perpendicular (parallel) to the beam axis. The horizontal dotted lines represent the unshifted position of the Zeeman sublevels. The shift results from the external magnetic field.

Both the limitations of range and alignment sensitivity can be addressed through the use of all-optical interrogation techniques that utilize electromagnetically induced transparency (EIT). These measurements eliminate the need for an external coil system by probing an atomic resonance structure that is fundamentally sensitive to changes in both the magnitude and direction of the magnetic field vector. The measurements performed with ^87^Rb atoms, with the energy level structure shown in figure 1, allow for the observation of seven transmission peaks associated with the available two-photon resonances between Zeeman sublevels. Measurements of the separation between these transmission peaks allow for scalar measurements of the absolute magnetic field. Changes in the direction of the magnetic field result in a variation of the coupling strengths between different magnetic sublevels, meaning that the direction of the field can be determined by examining the variation of the relative amplitudes of the transmission peaks. Therefore, measurements of the position and relative amplitudes of the EIT resonance peaks affords a means to perform vector measurements of the local magnetic field. This method for optical vector atomic magnetometry was developed theoretically [23] and demonstrated as a proof of concept [24].

**Figure 2.**
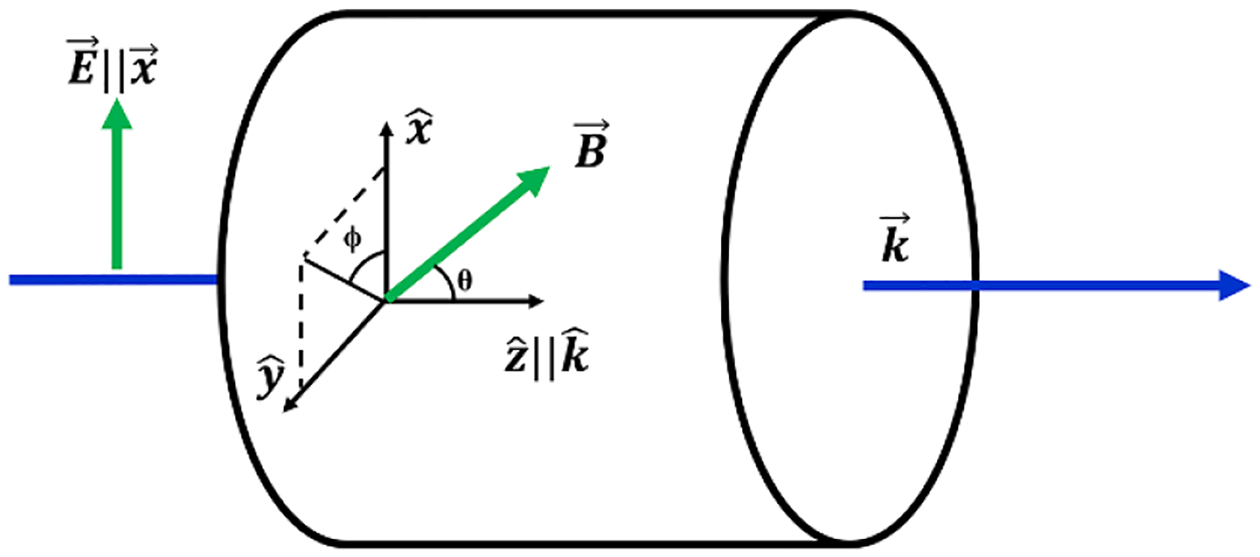
Magnetic field vector angle definitions used for the EIT wave vector $\vec{k}$. The laser coordinate system is defined by the laser beam direction and the polarization of the light. The angle ϕ denotes the azimuthal angle of the magnetic field and θ denotes the longitudinal angle.

**Figure 3.**
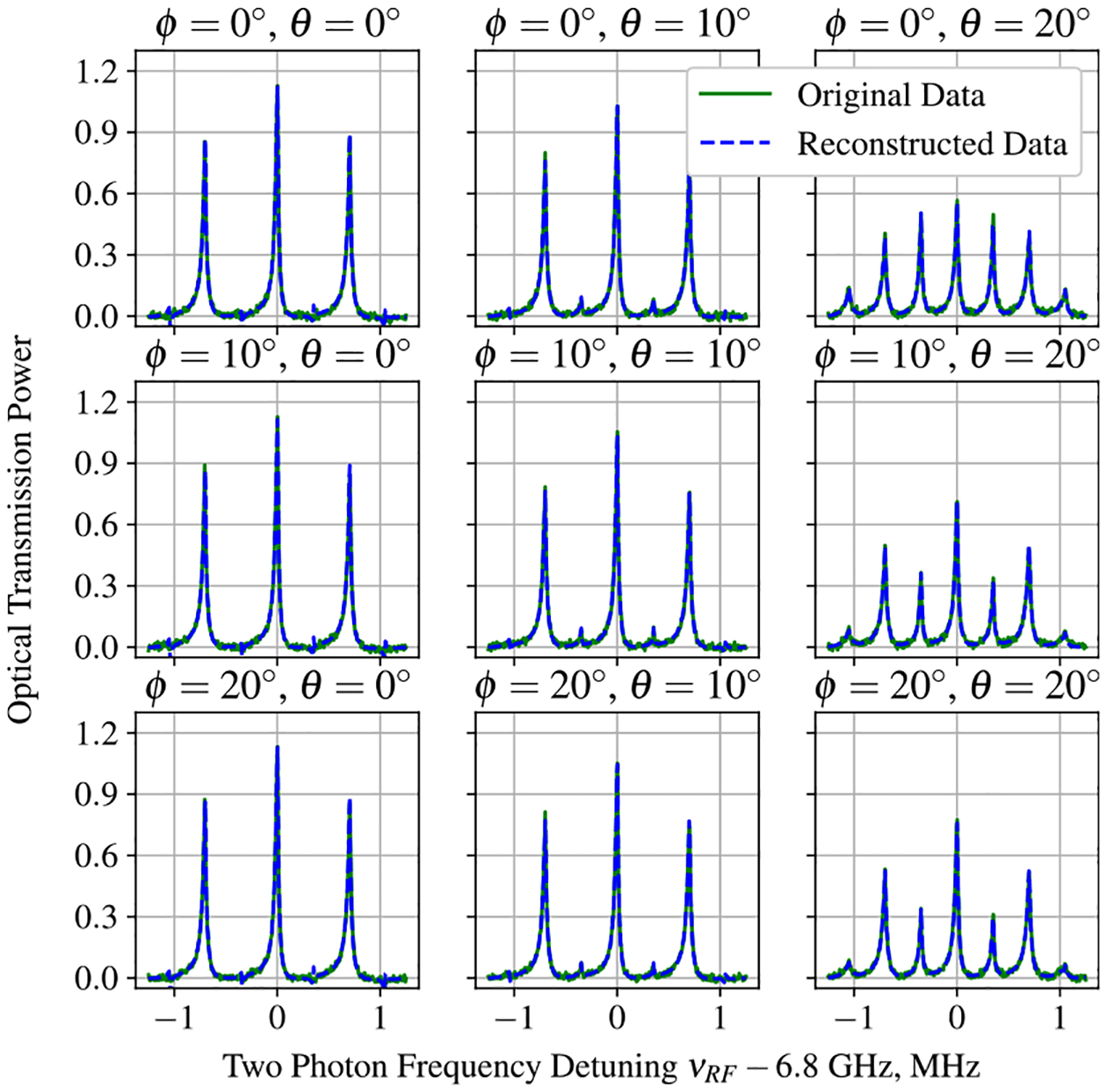
Sample EIT spectra recorded for different values of ϕ and θ. Measurements are performed by passing a laser beam through the Rb vapor cell and using a photodetector to record the transmission amplitude through the cell as a function of the laser detuning frequency. Measurements from experiment are shown with the green trace and examples of reconstructed EIT spectra using ten principal components are shown with the dashed blue trace. These spectra were reconstructed via the PCA technique discussed in the paper.

While the vector EIT magnetometer has relatively low maximum achievable sensitivity when compared against state-of-the-art scalar magnetometers, the EIT measurement approach overcomes many of the limitations associated with conventional vector magnetometers. The advantages of the EIT measurement strategy include acceptable accuracy of scalar measurements due to the measurement of the electron spin precession frequency, as well as direction determination based on a coordinate system entirely defined by the direction of the laser wave vector and the direction of the input polarization of light. These parameters are much simpler to align with an external coordinate system than the complex coil systems associated with alternative vector magnetometry methods. Furthermore, the EIT measurement approach allows for sensitivity over a large dynamic range (up to 1 T), overcoming the limited range capabilities of competing magnetometers. A diagram defining the vector coordinate angles is shown in figure 2. Given this cylindrical coordinate system, the magnetic field vector, $\vec{B}$, can be defined with the following expression:

(1) $$\vec{B}=|B|sin(\theta )cos(\phi )i+|B|sin(\theta )sin(\phi )j+|B|sin(\theta )k$$

where |B| is the magnitude, ϕ is the azimuthal angle, and θ is the longitudinal angle for the magnetic field. Examples of EIT spectra measurements recorded for different values of ϕ and θ are shown in figure 3.

While ϕ could be locked to the $\phi =0^{\circ }$ position by actuating the polarization of the lasers (it can be done in an experiment using an eclectically actuated liquid crystal polarization rotator), a method for identifying the θ is less trivial. Initially demonstrated in [24], it was shown that the information needed to recover θ is contained within the amplitudes of the seven EIT resonances (figure 3). Theoretically, θ can be determined by evaluating the ratios of specific resonances in the EIT spectrum. However, it was unclear if the measurement can accurately recover θ in a realistic physical system where shapes of the resonances are distorted. Furthermore, while the information about the magnetic field is contained in each of the resonances, it was unknown which ratios of amplitudes could optimally determine θ in atomic cells containing buffer gases. To address this problem, we explored a variety of unsupervised machine learning techniques to capture the empirical behavior of this system. The adoption of unsupervised learning techniques was motivated by a desire to assess the system’s behavior without relying on assumptions or detailed theoretical models. We found that dimensionality reduction techniques such as principal component analysis (PCA) were particularly effective at describing changes in the EIT spectra, and kernel PCA (KPCA), a nonlinear adaptation of PCA, was ultimately selected as the optimal procedure for determining θ in this type of EIT-based vapor cell vector magnetometer.

PCA methods can be used to reduce the dimensionality of the EIT spectra such that each particular spectrum can be represented as a coordinate in a new reduced dimensional subspace. The resulting coordinates can then be regressed to capture correlations between different configurations of the EIT spectrum and the direction of the local magnetic field. To this end, dimensionality reduction is not the primary objective in the application of KPCA to vector magnetometry. Rather, KPCA is used for feature extraction in service of the ultimate goal of recovering labels of ϕ,θ, and |B| from the observations of EIT spectra.

Applications of classical PCA techniques have been validated in quantum optics and used to study and classify phase transitions for muon spectroscopy [25], and the utility of nonlinear PCA methods in regression has also been thoroughly studied [26]. However, the potential of KPCA for regression problems in AMO spectroscopy has not been fully explored. While this paper specifically outlines the application of PCA to the EIT spectroscopy associated with this vector atomic magnetometer, the methods explained herein can be applied to any situation in which a user seeks to efficiently extract some complex relationship from a library of RF spectra.

Our experiments demonstrate that is is possible to create a vector magnetometer without involvement of the theoretical description of the physical process. The machine learning technique allows for measurement of the magnetic field with reasonable accuracy. This paper is organized as follows: in section II, we discuss the mathematics associated with applying variations of PCA to RF spectra; in section III, we apply these PCA methods to spectra collected with a prototype of the vector EIT magnetometer; in section IV, we demonstrate how the principal component projections can be utilized to recover individual scalar components of the magnetic field vector; in section V, we compare the PCA approach against a more conventional spectroscopy method and discuss how the PCA approach can be extended to recover additional components of the magnetic field vector; section VI concludes the paper.

## 2. Methodology

The central idea of the dimensionality reduction technique is to identify a limited number of eigenfunctions describing the possible variety of the EIT spectra, and then to use these eigenfunctions as a basis for a decomposition of the realizations of the spectra. As such, the decomposition coefficients will characterize modifications of the shape of the spectra. This approach is similar to the usage of Fourier decomposition in the harmonic spectral analysis, with the difference that PCA explicitly identifies the components experiencing maximum change with the variation of external parameters. In what follows we describe the approach in more detail and consider a few of its variations.

### 2.1. Principal component analysis

Classical PCA is the most widely used method of dimensionality reduction, and it is particularly effective for situations in which data is linearly separable [27]. Many real-world data sets are not linearly separable, and so nonlinear extensions of PCA have long been a source of interest within the study of dimensionality reduction [28, 29]. While this work explores the application of the nonlinear KPCA for magnetometry, the details concerning the implementation of KPCA are best understood first through the lens of classical PCA. A brief outline of PCA is provided below prior to the outline of the KPCA algorithm implemented for the magnetometer.

Let X be an n×p input data matrix containing n rows of independent observations and p columns of features. For the case of spectroscopic data, let us consider n as the number of spectrum measurement repetitions and p as the transmission values recorded at particular frequencies of the spectrum (number of points per spectrum). Thus X can be treated as a set of n,p-dimensional vectors in $R^{p}$. We wish to represent the high dimensional structure of these spectra in a more concise manner, so that X transforms to a set of n,m-dimensional vectors in $R^{m}$ (and m<p). Let us characterize possible variations of these spectra in terms of deviation from some average shape, the motivation for this step will be made clear later on.

Assuming the spectra have been aligned in frequency, we can define the mean spectrum by [30]

(2) $$\overset{‾}{X}=\frac{1}{n}\sum_{i=1}^{n}X_{i}.$$

Each observation of the spectrum, $X_{i}$, can then be described by the deviation from the mean spectrum $\overset{‾}{X}$, giving rise to the centered data matrix

(3) $$X_{0_{i}}=X_{i}-\overset{‾}{X},i=1\ldots n$$

where $X_{0_{i}}$ denotes the $i^{\text{th}}$ column of the centered data matrix $X_{0}$. The goal of PCA is to create a linear map that transforms $X_{0}$ into a new feature space $X_{0}\mapsto WX_{0}$, where W is the compression matrix of size p×m. Note here that the matrix multiplication is read from right to left. Projections of $X_{0}$ in the new feature space can be calculated with:

(4) $$F=WX_{0}$$

where the feature matrix F, with size n×m, is a representation of each observation in $X_{0}$ with the reduced dimensionality of m features. The loss associated with this compression process can be determined with some recovery matrix, R, with size m×p, such that an approximation of the original data matrix, ${\tilde{X}}_{0}$, can be calculated with ${\tilde{X}}_{0}=RWX_{0}$. As such, the central problem in PCA is to determine W and R in a manner that minimizes the total squared Euclidean distance between the original and reconstructed data matrices. This is stated mathematically in equation (5) [31]:

(5) $$\underset{W,R}{argmin}\sum_{i=1}^{n}{∥X_{0_{i}}-RWX_{0_{i}}∥}^{2}.$$

#### 2.1.1. PCA via covariance matrix

Previous studies have demonstrated that the objective of minimizing the reconstruction error is equivalent to maximizing the variance of the projected data [32], and both can be accomplished through eigendecomposition of the sample covariance matrix of X, given by:

(6) $$C=\frac{1}{n}X_{0}^{T}X_{0}.$$

Equation (6) results in a p×p matrix where the diagonal entries describe the variance between features of X and all other entries describe the covariance between features of X. Therefore, the total variance of X can be calculated by taking the trace of C. The corresponding eigenvalue expression for matrix C is:

(7) $$Cu_{k}=\lambda_{k}u_{k},k=1\ldots p$$

where $\lambda_{k}$ is an eigenvalue of C corresponding to the eigenvector $u_{k}$, and $u_{k}^{T}u_{k}=1$. The eigenvectors $u_{k}$ are therefore an orthonormal set of representative EIT spectra into which any measured spectrum can be decomposed. Vectors from the centered data set may be projected onto the orthonormal basis defined by these eigenvectors with

(8) $$X_{\text{proj}}=AX_{0}$$

where $A={\left[u_{1}\ldots u_{p}\right]}^{T}$ is the matrix of eigenvectors of C. The covariance of $X_{\text{proj}}$ then is given by:

(9) $$C_{\text{proj}}=\frac{1}{n}X_{\text{proj}}^{T}X_{\text{proj}}=\frac{1}{n-1}A^{T}CA$$

Because the vectors of A are orthonormal, this mapping is a similarity transformation and therefore $X_{\text{proj}}$ preserves the total variance of X.

It is now possible to reconstruct individual vectors in the centered data set as a linear combination of the projections and eigenvectors:

(10) $$X_{0_{i}}=\sum_{k=1}^{p}u_{k}\left(u_{k}^{T}X_{0_{i}}\right).$$

The eigenvectors of C are called the principal axes (also known as principal directions) of our data set, and the projections of specific observations onto these axes are called the principal components. Note however that this nomenclature can be inconsistent in the literature, and that some sources use ‘principal components’ to refer to the eigenvectors themselves rather than projections onto these eigenvectors. For the purposes of this paper, the term ‘principal components’ refers exclusively to the projections of X into the reduced dimensional subspace. Recall that we calculated the principal components and axes of X using the centered data matrix $X_{0}$. The analysis can also be performed with the original, non-centered data matrix X. However, under this approach, the first principal axis of the data will simply be the average shape of the input data. Ordinarily, the average shape is not particularly insightful in evaluating patterns within the data set, and so it is often discarded by first centering the input data.

The eigenvalues of C correspond to the amount of explained variance described with each eigenvector. If we select only m eigenvectors corresponding to the largest m eigenvalues, we can approximate $X_{0_{i}}$ with equation (11):

(11) $${\tilde{X}}_{0_{i}}=\sum_{k=1}^{m}u_{k}\left(u_{k}^{T}X_{0_{i}}\right).$$

This expression is equivalent to ${\tilde{X}}_{0}=D^{T}DX_{0}$, where $D={\left[u_{1}\ldots u_{m}\right]}^{T}$ has been constructed as the matrix of eigenvectors corresponding to the m largest eigenvalues. Therefore, equation (5) is satisfied if we set W to D and R to $D^{T}$. Any future observations of the system, $X_{\text{new}}$ may also be projected onto the principal axes with:

(12) $$X_{{\text{proj}}_{\text{new}}}=D\left(X_{\text{new}}-\overset{‾}{X}\right).$$

#### 2.1.2. PCA via Gramian matrix

The above solution assumes that n>p for the calculation of D. In the event that p>n, it is computationally more efficient to compute the Gramian matrix of X in place of the covariance matrix. While the covariance matrix (size p×p) describes the Euclidean distance between the different features of X, the Gramian matrix (size n×n) describes the Euclidean distance between the different observations of X. The Gramian matrix takes the form:

(13) $$G=\frac{1}{n}X_{0}X_{0}^{T}.$$

Once again, PCA is performed by solving the eigenvalue problem for G and retaining only the m eigenvectors corresponding to the m largest eigenvalues. However, note that under this formulation, the eigenvectors of G do not correspond to the principal axes of X. Instead, eigenvectors of G correspond to the principal component projections of X. The cause of this difference is clear when PCA is considered through the framework of single value decomposition (SVD) [33]. The SVD theorem states that a real matrix can be represented by [34]:

(14) $$X_{0}=U\Sigma V^{T}$$

where U is an n×n orthonormal matrix where the columns are the eigenvectors of $X_{0}X_{0}^{T}$ and V is a p×p orthonormal matrix where the columns are the eigenvectors of $X_{0}^{T}X_{0}.\Sigma$ is an n×p diagonal matrix of the singular values of $X_{0}$, which are given by the square roots of the eigenvalues of either $X_{0}X_{0}^{T}$ or $X_{0}^{T}X_{0}$. Note that the eigenvalues of $X_{0}X_{0}^{T}$ and $X_{0}^{T}X_{0}$ are equivalent. By plugging equation (14) into equation (6), we get:

(15) $$C=V\frac{\Sigma^{2}}{n}V^{T}.$$

This expression is simply the eigendecomposition of C, and it affirms that the SVD matrix V coincides with the matrix A. By extension, the UΣ term in equation (14) coincides with the principal components of X. Similarly, by plugging equation (14) into equation (13), we have:

(16) $$G=U\frac{\Sigma^{2}}{n}U^{T}.$$

So we see that the eigenvectors of G coincide with the matrix U in equation (14). Because Σ is only a diagonal matrix of the singular values of X, we can see that the matrix U also coincides with principal components of X. However, these projections will not be scaled by the singular values of X, as they would be with the covariance method. In order to calculate the principal components of X using the Gramian method such that they coincide with the principal components of the covariance method, the eigenvectors of G must be scaled by the square roots of the eigenvalues (the singular values of X):

(17) $$X_{{\text{proj}}_{G}}=U\Sigma .$$

The computational complexity associated with solving PCA via the covariance matrix is $O\left(np^{2}\right)$ for the construction of C plus $O\left(p^{3}\right)$ for calculating the eigenvalues of C. The computational complexity of solving PCA with the Gramian matrix is $O\left(n^{2}p\right)$ for the construction of G plus $O\left(n^{3}\right)$ for calculating the eigenvalues of G. Therefore, the decision of performing PCA through the covariance or Gramian matrix should be determined by the relative size of the observations and features of the data set.

### 2.2. Kernel principal component analysis

Note that the Gramian matrix method allows us to calculate U without the need to explicitly calculate V. This means that for a given data set, only the inner products between vectors need to be calculated in order to perform PCA. KPCA exploits this attribute by mapping the input data into a higher dimensional space and computing the Gramian matrix implicitly. While this step may seem counter intuitive to the goal of dimensionality reduction, it is worth noting that data that is not linearly separable can often be made linearly separable in a higher dimensional space. KPCA involves projecting the input data into this high-dimensional space and then applying classical PCA to the projected data via the Gramian matrix method. This nonlinear dimensionality reduction allows us to obtain a reduced dimensional representation of the data that is linearly separable, even when the input data is not.

The process is accomplished by introducing a nonlinear transformation ϕ(x) that maps the p-dimensional vectors of X into an ℓ-dimensional feature space where ℓ>p (let us assume for the moment that $X_{0}$ remains centered under this mapping, see equation (3)). If ℓ is not too large, PCA could still be performed explicitly in the higher dimensional space with the covariance matrix:

(18) $$C=\frac{1}{n}\phi {\left(X_{0}\right)}^{T}\phi \left(X_{0}\right).$$

This calculation will now have the computational cost of $O\left(n\ell^{2}\right)$ for the construction of C plus $O\left(\ell^{3}\right)$ for calculating the eigenvalues of C. Fortunately, kernel substitution can be utilized to calculate the Gramian matrix of the ℓ-dimensional data using only the original p-dimensional data [28]. Kernel substitution consists of defining an arbitrary kernel function (such as the nonlinear transformation described above) that is used to calculate an n×n kernel matrix for X. The entries of the kernel matrix can be expressed in terms of pairs of the p-dimensional input data vectors. The n×n kernel matrix, expressed in terms of the kernel function ϕ(x) is given by:

(19) $$k\left(X_{i},X_{j}\right)=\phi \left(X_{i}\right)\phi {\left(X_{j}\right)}^{T}.$$

While we initially assumed that $X_{0}$ remained centered under ϕ(x), this cannot be assumed to be true in general. In order to avoid the cost of computing the ℓ-dimensional average shape vector, we can instead determine the centered kernel matrix with the following expression [32]:

(20) $$\tilde{K}=K-1_{n}K-K1_{n}+1_{n}K1_{n}$$

where $1_{n}$ is an n×n matrix where each element is 1/n. $\tilde{K}$ is then equivalent to the Gramian matrix of the ℓ-dimensional data, and the eigenvectors of $\tilde{K}$ denote the nonlinear principal components of X scaled by the singular values of X.

An assortment of kernel functions can be selected for KPCA. Popular kernels include polynomial, sigmoid, and radial basis function (RBF) kernels. For the EIT spectra evaluated within this paper, a Gaussian RBF kernel was chosen, with the associated kernel matrix shown in equation (21). This kernel function was selected because of the flexibility in nonlinear mapping provided by the hyperparameter γ, which is included in equation (21) as a free parameter to be optimized for a specific application

(21) $$k\left(X_{0_{i}},X_{0_{j}}\right)=\text{exp}\left(-\gamma {∥X_{0_{i}}-X_{0_{j}}∥}_{2}^{2}\right).$$

The value of γ can be understood qualitatively as the extent to which the resulting dimensionality reduction is nonlinear, and the selection of γ is typically determined through unsupervised clustering methods or supervised cross validation methods. Guidelines for selecting an appropriate value of γ with both approaches are discussed in sections 3 and 4 of this paper. For the EIT magnetometer, varying γ was found to be an effective method of adjusting the clustering of the EIT spectra projections. The nonlinearity introduced with KPCA allowed the magnetometer model to resolve subtle changes in the EIT spectra, which were found to be associated with subtle changes in the magnetic field. Outside of magnetometry, some excellent examples of KPCA applications can be found in [35–37].

### 2.3. Regression

While this work is focused on exploring the potential of PCA methods for feature extraction of EIT spectra, creating an operational magnetometer that utilizes PCA for vector measurements requires the implementation of a regression model that correlates these features back to physical quantities associated with the observed magnetic field. The problem becomes a supervised learning task, requiring data sets in which the true values of $\vec{B}$ are known for each EIT spectrum. The appropriate regression algorithm to select depends heavily on the system behavior observed with PCA, but for the EIT magnetometer considered in the study, support vector regression (SVR) machines were found to efficiently fit the observed trend of principal components. The SVR approach was selected because it was flexible and well-suited to the inherent sparsity of the EIT data set (each spectrum is associated with discrete values of the magnetic field, but ϕ and θ are truly continuous values).

The goal of the SVR machine is to identify some function, f, that can approximate the data such that the difference between the predicted values and true values lie within some preset threshold, ϵ. The function is calculated so that the prediction error is within the ϵ limit for as many data points as possible while keeping the function as flat as possible. Similar to KPCA, the SVR method accomplishes this goal by employing a kernel trick to project the input data into a higher dimensional feature space. In the new feature space, the projected data is fit to a maximum-margin hyperplane in which linear regression can be performed. A detailed explanation on the implementation of the SVR machine is beyond the scope of this paper, but an excellent overview can be found with [39–41]. Additionally, examples of SVR incorporated with KPCA can be found in [42, 43]. For the EIT magnetometer, the SVR machine was trained to receive principal components calculated from the EIT spectra as an input, and would then make a continuous-valued prediction for the scalar quantities of θ,ϕ, or B associated with the measured spectrum.

## 3. Application

**Figure 4.**
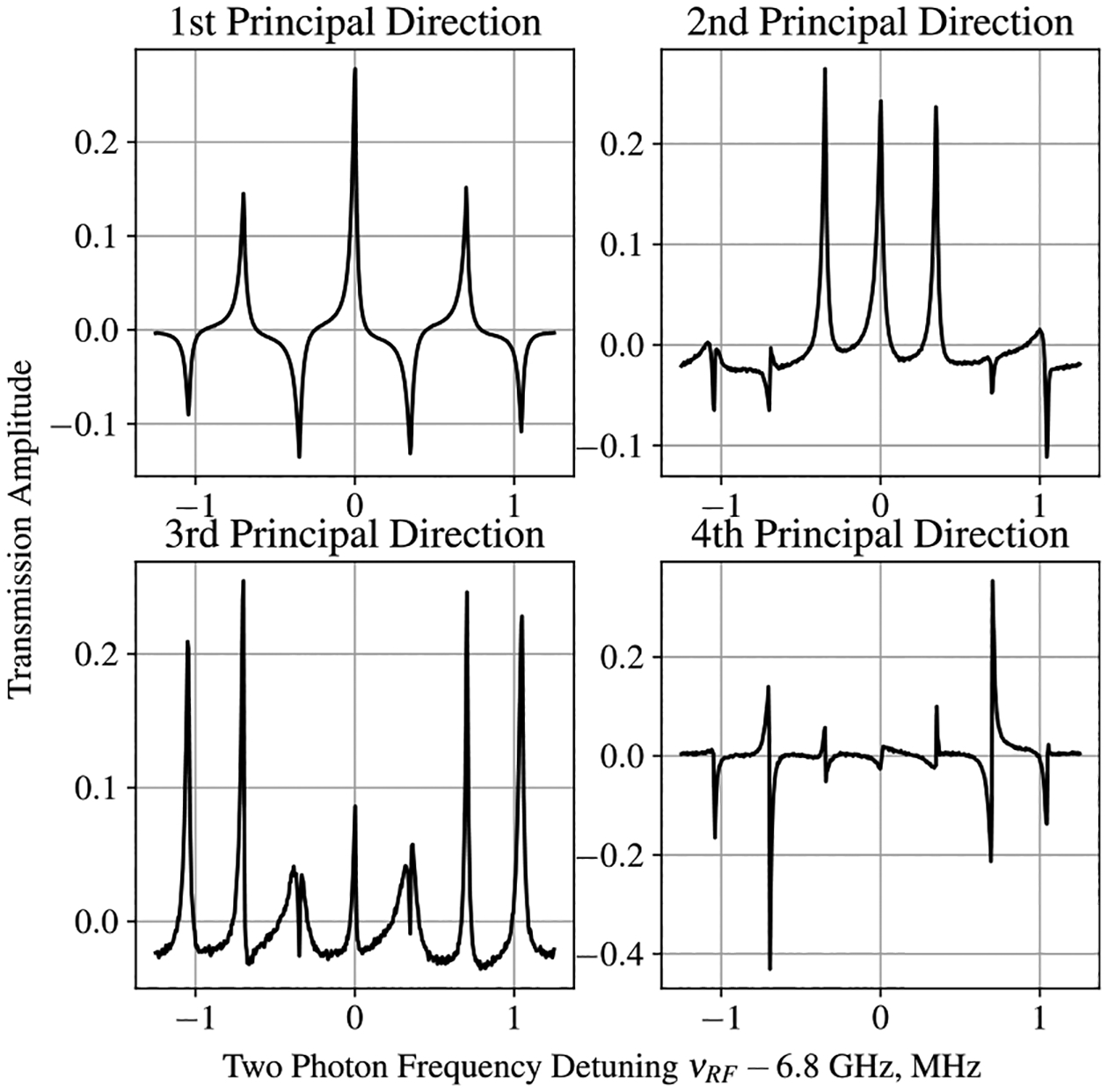
First four principal axes calculated for the EIT data set. Projections of measurement data onto this basis (the principal components) describe the extent to which these shapes are present in the original measurements.

**Figure 5.**
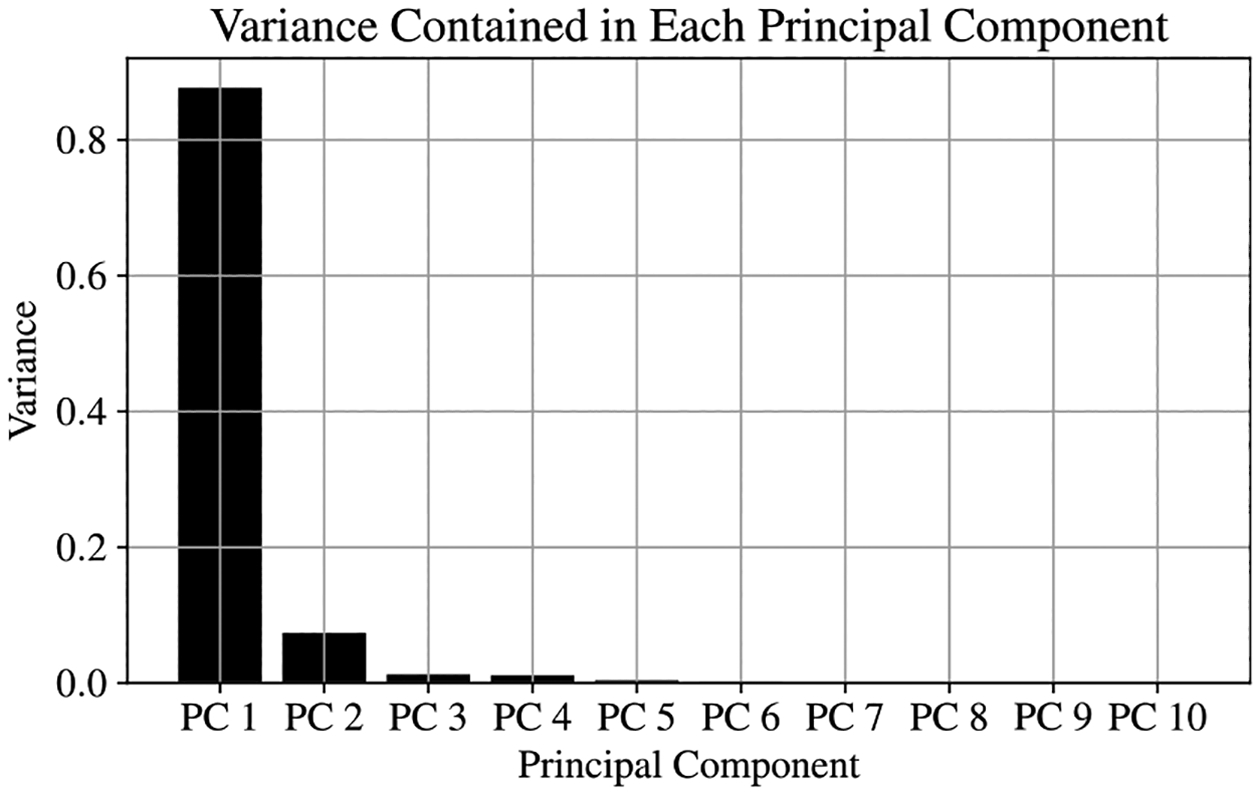
Eigenvalues calculated from covariance matrix of the EIT spectra. Over 98% of the total variance of the data set can be explained using only the first ten principal components.

To explore the relationship between EIT spectra and the direction of the magnetic field, the EIT Rb magnetometer was tested in a magnetically shielded environment. Manipulating the current in the Helmholtz coils, placed inside the shields, adjusted the direction of the applied magnetic field. For the initial testing, the applied field was maintained with an approximate magnitude of 500 mG (Earth’s field). The EIT spectrum was recorded with 10° increments in the azimuthal angle, ϕ, and 5° increments in the longitudinal angle, θ, with multiple spectra recorded for each direction. Each spectrum consisted of 500 transmission amplitudes measured as a function of the laser detuning frequency, and approximately 1500 spectra were collected for the initial evaluation. The EIT measurements were prepared into the format listed in section II, creating a 1500 × 500 data matrix on which PCA could be performed. After calculating the mean spectrum and centering the data, the 500 × 500 covariance matrix was assembled to perform classical PCA on the EIT data set. The first four principal axes of the EIT data set, corresponding to the eigenvectors of the covariance matrix with the four largest eigenvalues, are shown in figure 4. The relative explained variance contained in the first 10 eigenvectors is shown in figure 5.

**Figure 6.**
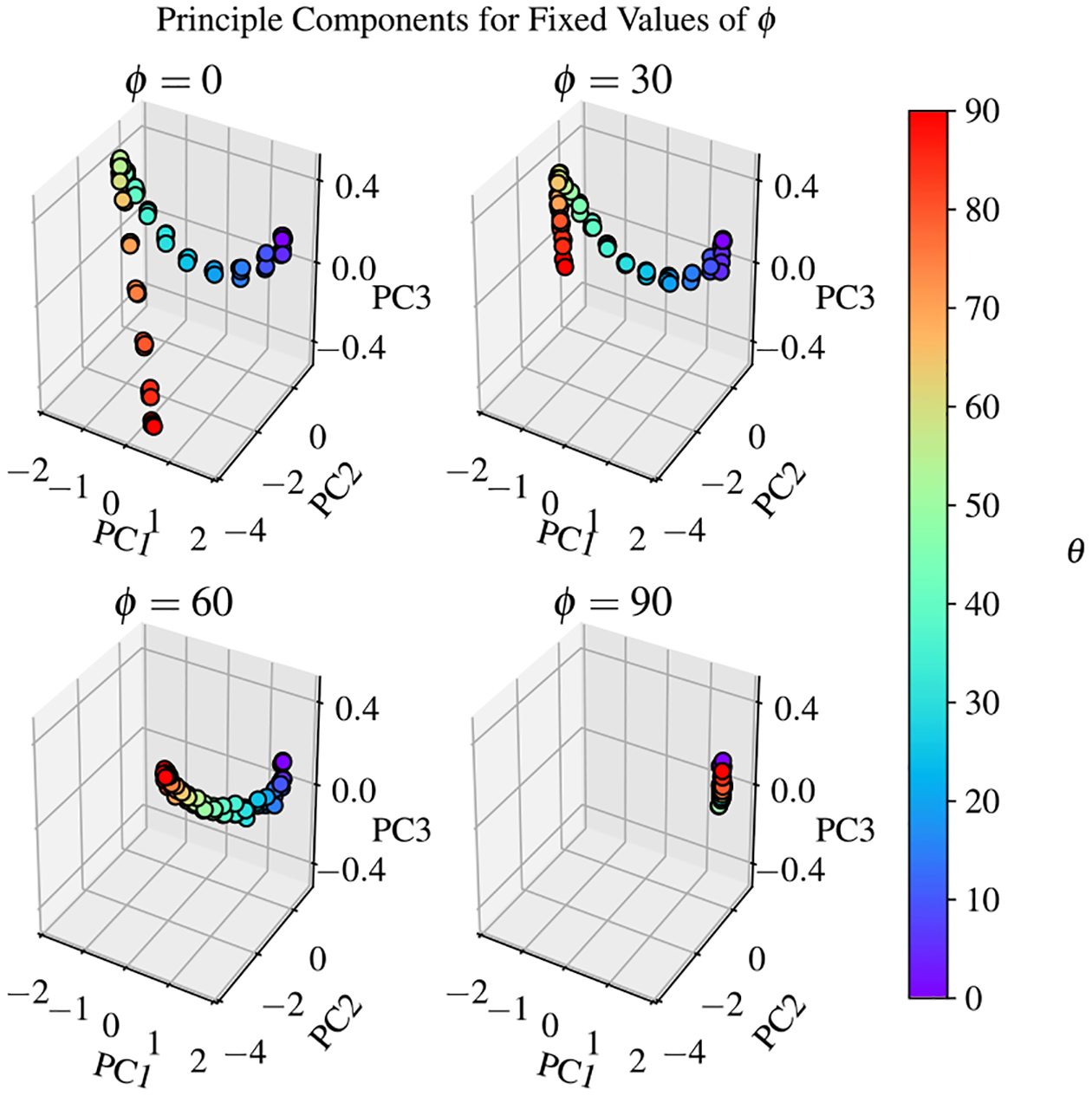
Scatter plots of principal component projections for measurements within the EIT data set, grouped together by values of ϕ. The color gradient denotes the value of the longitudinal angle, θ, for each measurement. Each cluster corresponds to some ϕ and θ. The size of the cluster characterizes measurement error. The least error is achieved for ϕ=0° and the largest error is observed in the case of ϕ=90°.

An approximation of the original data set can be calculated using a linear combination of the principal components and axes. Examples of the reconstructed EIT spectra are shown in figure 3. While this reconstruction process can be useful for denoising a signal, the true utility of PCA is found in its ability to represent each measurement as a coordinate in the reduced dimensional space. Figure 6 shows the first three projections of each measurement for the EIT data set in a three dimensional scatter plot for different values of the azimuthal angle ϕ.

Classical PCA provides a means to quantify the sensitivity of this measurement system with respect to the determination of θ as a function of ϕ. As figure 6 shows, the principal components of the EIT spectra all fall along a smooth curve in the reduced dimensional space. Note that in the top left plot of figure 6, we observe clustering at specific values of θ. This trend continues for each value of ϕ; however, at higher values of ϕ, the spacing between clusters decreases. Fortunately, ϕ can be independently controlled with the polarization of the laser. Therefore, for optimal performance, the laser polarization must be locked to $\phi =0^{\circ }$ in order to utilize the full range of possible amplitude variations in the EIT spectra.

**Figure 7.**
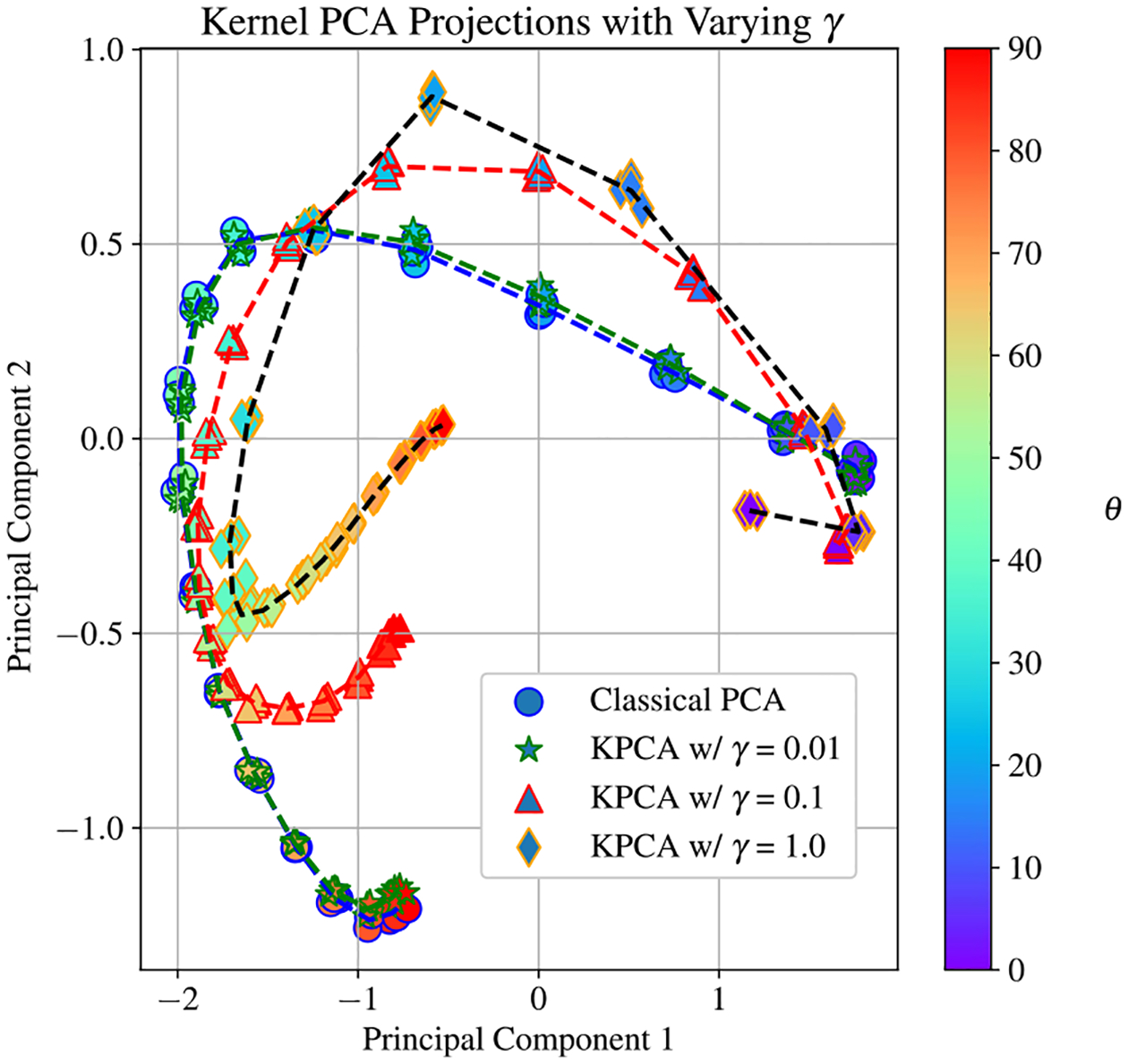
Scatter plot showing the first two principal component projections for each measurements where ϕ=0 for different values of γ for the KPCA RBF kernel. In this plot, all of the projections have been scaled by the singular values of the EIT data set As γ increases, the clusters corresponding to low values of θ become well resolved. For classical PCA, these same clusters are not well separated, and it is difficult to resolve small changes in the spectrum at low values of θ. This relationship illustrates the utility of the KPCA technique.

For KPCA, a similar trend can be observed, but the RBF kernel function introduces the tunable parameter γ that can adjust the contour of the projections curve. The influence of γ on the projections curve is demonstrated with figure 7. For low values of γ, the RBF kernel converges to a linear kernel, and so the classical and kernel principal component projections coincide. As γ increases, both the broader projections curve and the clustering behavior change. Thus, KPCA provides some flexibility in arranging how different measurements may be grouped together. This flexibility is particularly valuable in resolving small values of θ, in which the classical PCA projections are not well separated. Note that the trends shown here extend beyond the first three principal components, and the two and three dimensional scatter plots are only included to give the reader a qualitative understanding of the sort of system behavior that PCA methods can uncover.

**Figure 8.**
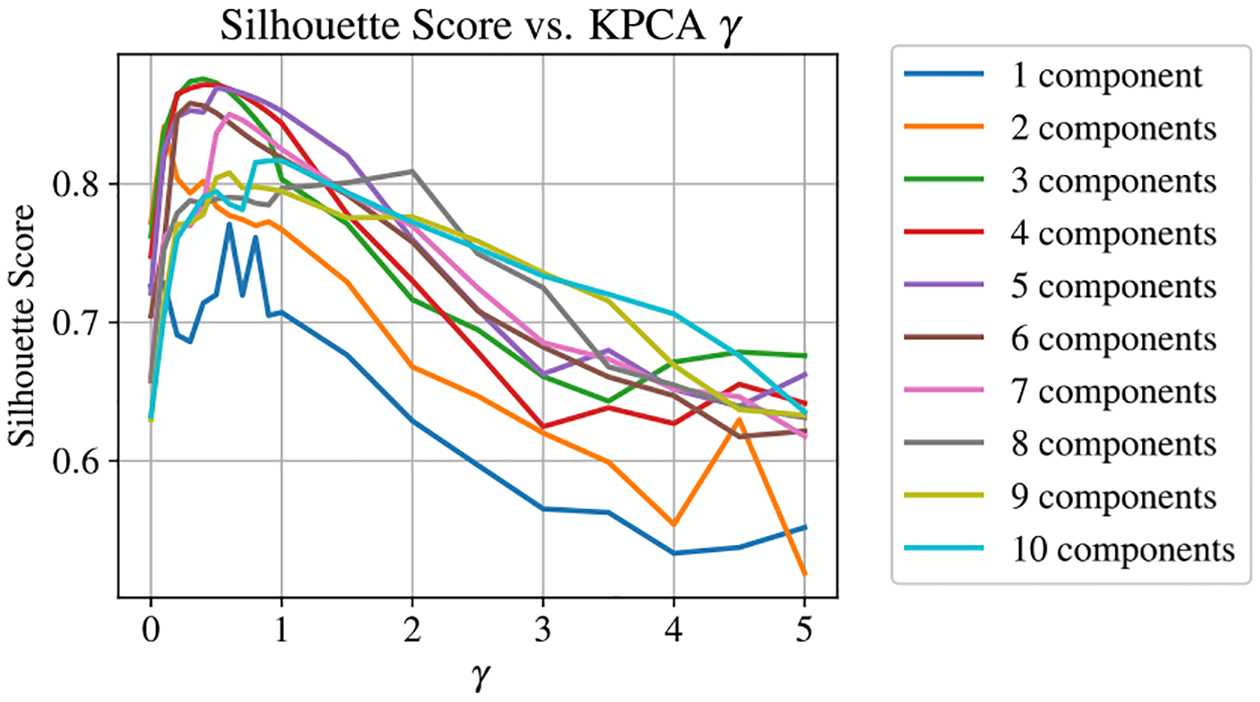
Influence of KPCA γ on the silhouette score, $C_{S}$ for the EIT data set.

For many applications, γ is optimized with cross-validation approaches, in which a regression model is created to evaluate the predictive power of the KPCA projections as a function of γ. Naturally, this method necessitates a supervised learning algorithm that requires assumptions about how different observations of the data may be correlated. Such assumptions potentially defeat the purpose of the unsupervised analysis, because the achievable performance of the model will be dependent on these underlying assumptions. Fortunately, there a clustering evaluation metrics that can be incorporated to optimize γ without compromising the unsupervised nature of the analysis. For the EIT dimentionality reduction, the silhouette score method was used to optimize γ as it pertains to resolving values of θ. The silhouette score was calculated by fitting the KPCA projections into k clusters with a k-means clustering algorithm and evaluating the size of clusters relative to the separation between neighboring clusters [38]. For a given set of KPCA projections, the silhouette score, $C_{S}$, was calculated using:

(22) $$C_{S}=\frac{1}{n}\sum_{i}^{n}\frac{b_{i}-a_{i}}{max\left(a_{i},bi\right)}$$

where $a_{i}$ is a given coordinate in the reduced dimensional feature space and $b_{i}$ is the closest coordinate from the neighboring cluster. For the EIT measurement where ϕ=0, there were 19 distinct values of θ measured. Thus, the k-means clustering algorithm was used to fit the KPCA projections into 19 clusters, and $C_{S}$ was evaluated as a function of both γ and the number of kernel principal projections used in the dimensionality reduction. The resulsts of this analysis are shown in figure 8.

KPCA achieves improved clustering over classical PCA for the higher order principal components at optimal values of γ. This can be seen in figure 8, as the low values of γ correspond to the clustering performance of classical PCA. This analysis suggests that the optimal performance of the model is found using 3–4 principle components and a γ≈0.3. The improved performance at higher values of γ demonstrates that some manifestations of the EIT spectra are not linearly separable, and so the kernel function is needed to resolve values of θ corresponding to these spectra.

## 4. Results

The above analysis highlights the value of unsupervised machine learning for gaining insight into the inner workings of the magnetometer system, particularly with the identification that the greatest variation in the EIT spectra occurred when the polarization ϕ was locked to 0°. In response to this finding, two additional data sets were collected to assess the performance of the EIT magnetometer using the PCA/KPCA and the supervised SVR algorithm. The first data set consisted of 2037 measurements of EIT spectra associated with a magnetic field where ϕ=0° and the absolute field B was maintained at 500 mG. This data set was used to characterize the ultimate sensitivity of the PCA method for directionality measurements. The second data set consisted of 1415 measurements of EIT sepctra where ϕ=0° and the absolute field was varying from 50 to 56μT, and this data set was used to characterize sensitivity of the PCA method for scalar measurements. Results from both modes of measurement are discussed in the following section.

### 4.1. Angular measurements

The 2037 measurements of EIT spectra were separated into a training group of 1425 samples and a test group of 612 samples. The training samples were used to calculate the principal components of the data set, and the SVR model was trained to recover specific values of θ using five-fold cross validation on the principal components of the 1425 training samples. Hyperparameters of the SVR model were optimized using the coefficient of determination, $r^{2}$, given by:

(23) $$r_{\theta }^{2}=1-\frac{\sum_{i}^{n}{\left(\theta_{i}-{\tilde{\theta }}_{i}\right)}^{2}}{\sum_{i}^{n}{\left(\theta_{i}-\overset{‾}{\theta }\right)}^{2}}$$

where $\theta_{i}$ is the observed (true) value of θ associated with the ith spectrum (controlled in experiment by applying fixed currents to a set of three-dimensional Helmholtz coils), ${\tilde{\theta }}_{i}$ is the predicted value of θ from the PCA-SVR model, and $\overset{‾}{\theta }$ is the average value of θ. The SVR machine also incorporated an RBF kernel for fitting the principal component curve, and it was implemented and optimized using various libraries from Scikit-Learn in Python [44].

**Figure 9.**
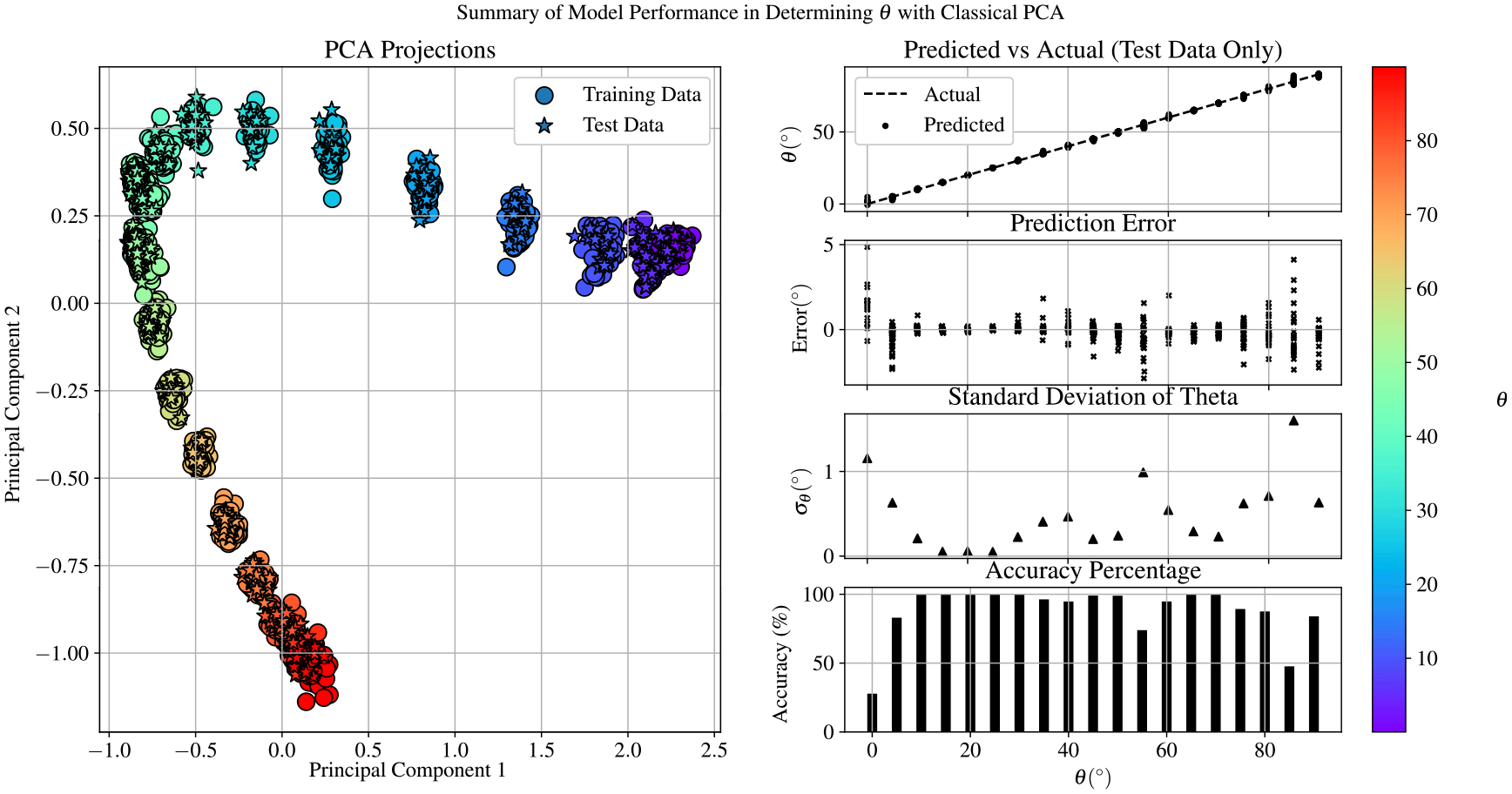
Summary of performance for the PCA-SVR model in recovering θ from EIT spectra. The SVR model was trained using three principal components. The scatter plot to the left depicts the principal components of all measurements incorporated in training, validating, and testing the PCA-SVR mode. The plots to the right depict the performance of the model using only the 612 samples from the test group.

The above training method was first used with classical PCA, and a summary of the PCA-SVR model performance is provided in figure 9. Note that here we define accuracy as the percentage of measurements in which spectra from the test data set were used to correctly predict the value of θ within one degree of the true value from experiment. Clearly the classical PCA method works reasonably well in predicting values of θ. However, this summary shows that the model struggles to resolve both lower and higher values of θ. There are no tuning parameters to be optimized with classical PCA, and so this is the best performance that this method can achieve.

**Figure 10.**
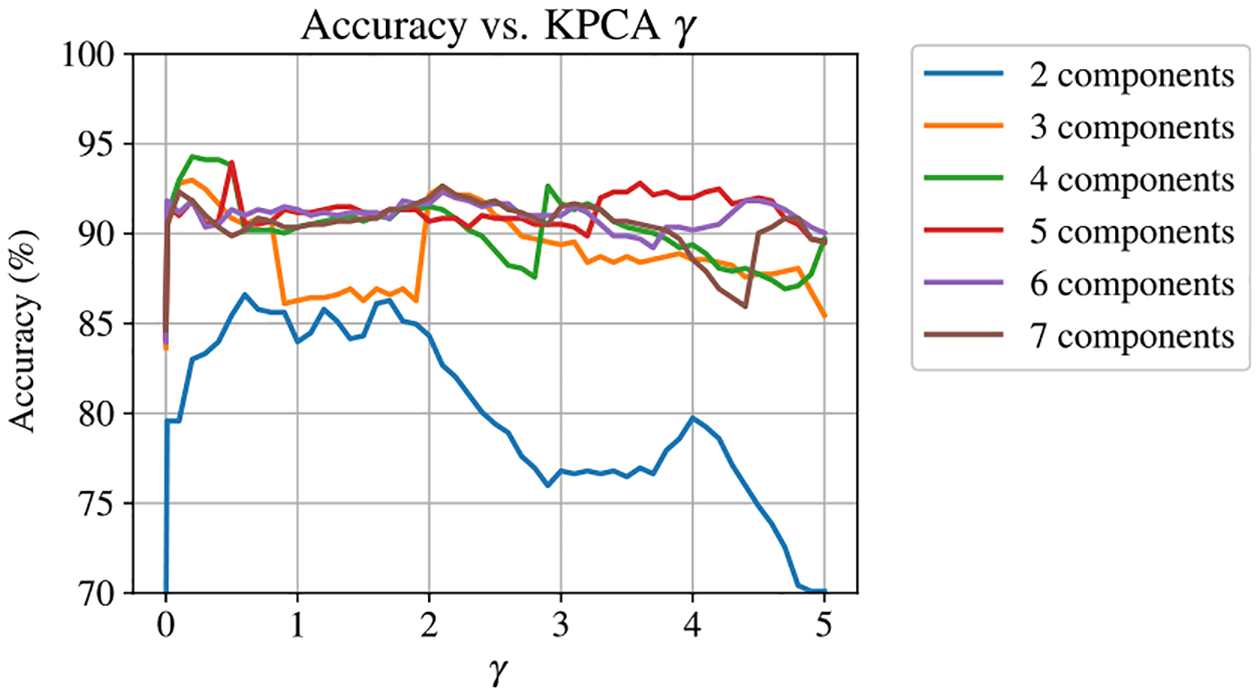
Influence of KPCA γ on the accuracy of the SVR model, with accuracy expressed in terms of the percentage of predictions within one degree of the true value of θ.

The training method was then repeated using the KPCA approach, with the additional step of optimizing γ for the KPCA RBF kernel alongside the hyperparameters of the SVR machine. The accuracy of the KPCA-SVR model evaluated as a function of KPCA γ is shown in figure 10. This result agrees well with the unsupervised analysis from figure 8, and once again we find that the KPCA model is optimized with four principal components and a γ≈0.3. Note that this plot also shows the accuracy of classical PCA as a function of the number of principal components for low values of γ.

**Figure 11.**
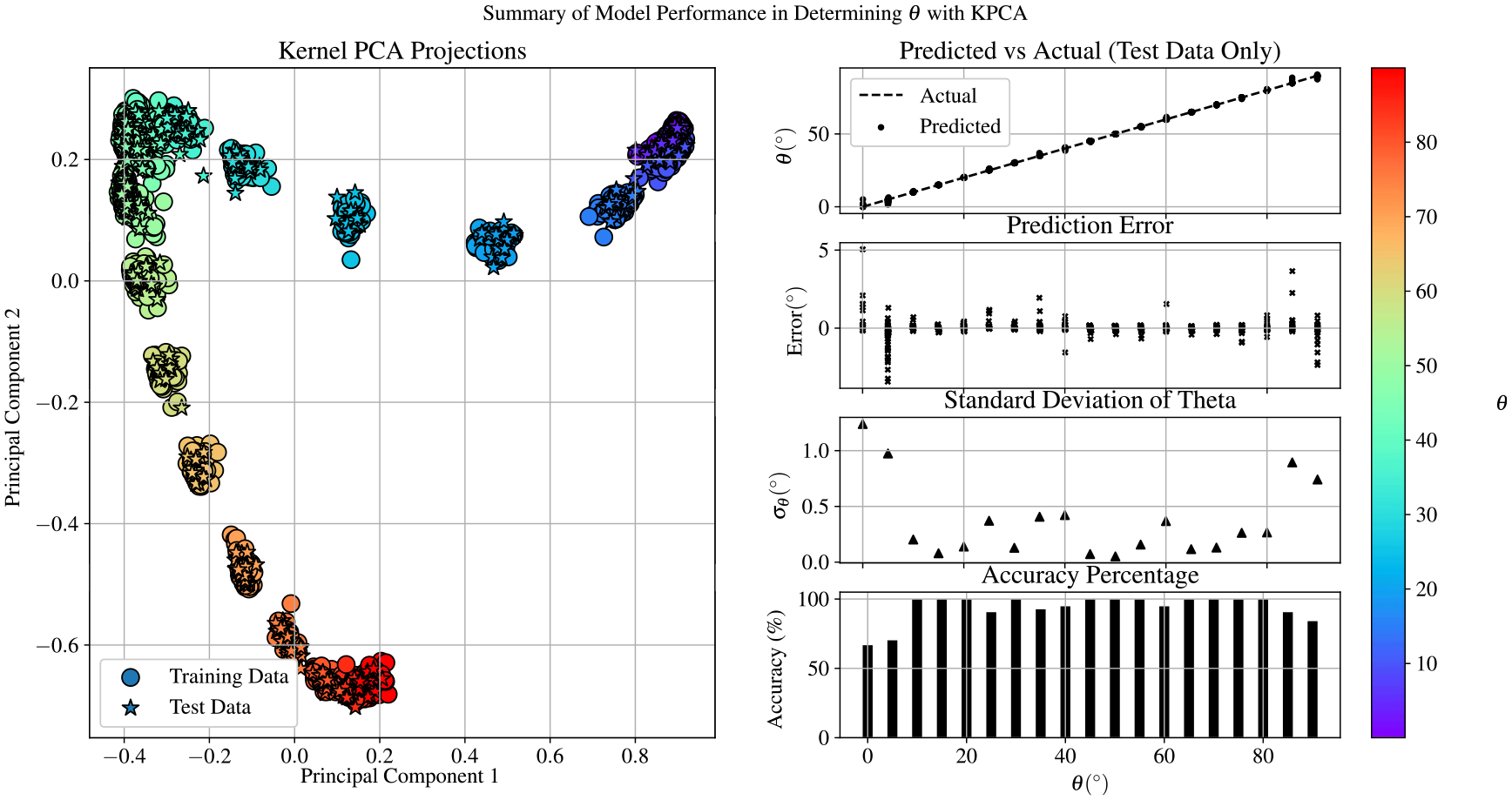
Summary of performance for the KPCA-SVR model in recovering θ from EIT spectra. The SVR model was trained using four kernel principal components calculated with an RBF kernel (γ=0.35).

Once optimized, the KPCA-SVR model was able to predict the value of θ for a given EIT measurement with a standard deviation better than 1 degree and an $R^{2}$ score of 0.9996 on the test data set. A summary of the performance of the KPCA-SVR model is shown in figure 11. Here we see that the nonlinear dimensionality reduction introduced with KPCA is able to resolve the the the extremities of θ, affirming that some manifestations of the EIT spectra are not linearly separable.

### 4.2. Scalar measurements

**Figure 12.**
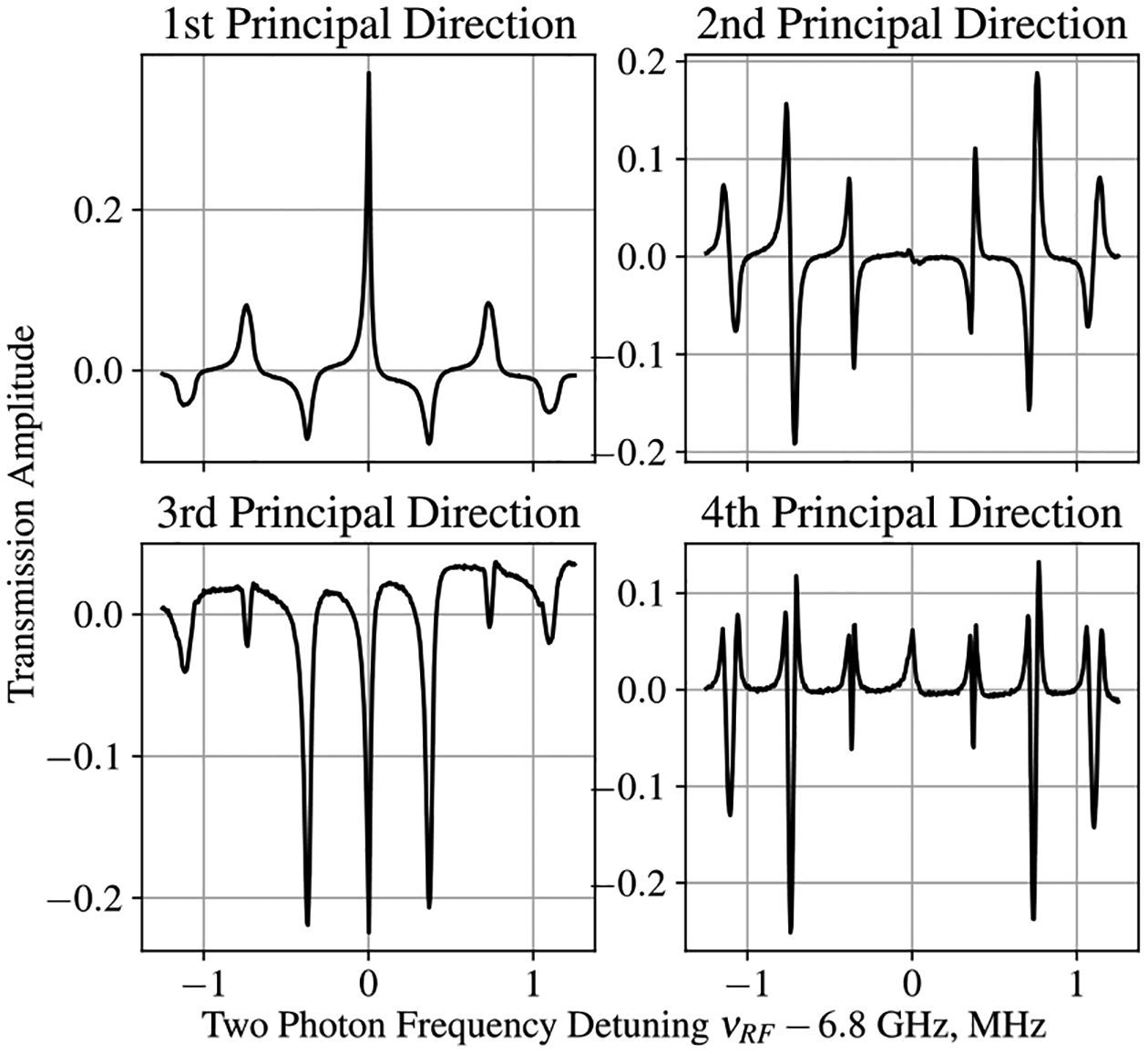
First four principal axes calculated for the EIT data set with variations in |B| from 50 to 56 μT. Note the line broadening of the first principal axis, resulting from variations of the position of the EIT resonances caused by different magnitudes of the absolute field.

Similar to the directionality measurement analysis, the 2537 measurements containing variations in θ and |B| were divided into 990 training samples and 425 test samples. These training samples were used fit the PCA model and train a new SVR model to predict the magnitude of $\vec{B}$ from the EIT spectra. Variations in the absolute field do not influence the line shape or the relative amplitudes of the EIT spectra, but they do change the separation between the transmission peaks. The principal axes calculated with this data set reflect this behavior, and we can see in figure 12 that the line shape of the first principal axis has been broadened when compared against that of the first principal axis shown in figure 4. Furthermore, the second principal axis closely resembles the first derivative of the EIT spectra, which is precisely the feature that would be used to make this scalar measurement in a classical laboratory environment.

**Figure 13.**
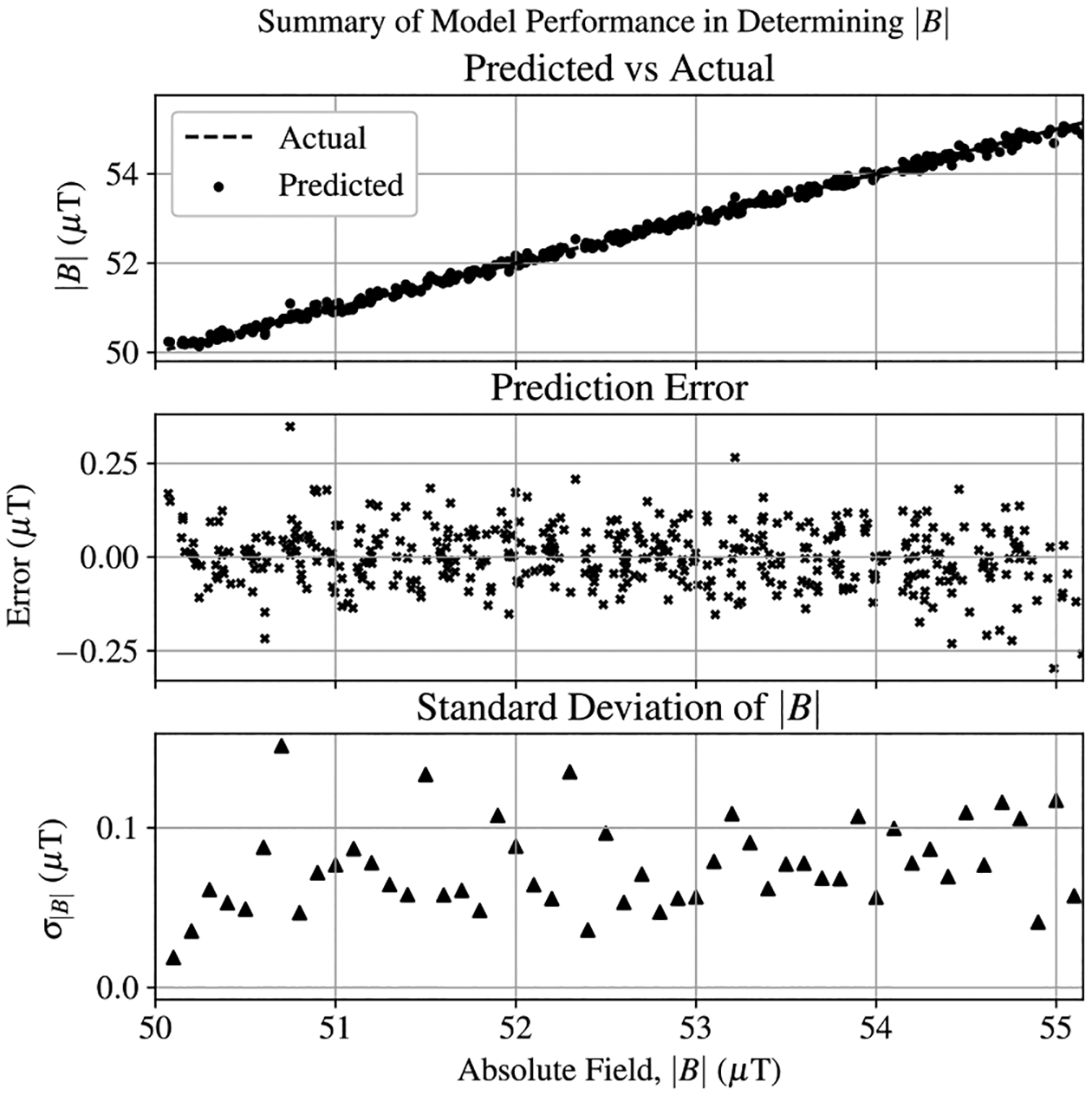
Performance summary of the PCA-SVR model in recovering |B| from EIT spectra.

The trained PCA-SVR model was used to predict |B| for the 425 test samples. This proved to be a very effective method of determining |B|, producing an average standard deviation of 70 nT across all values of |B|. The performance of this method is summarized in figure 13. This analysis was performed using only the classical PCA approach, assuming that a linear model would be sufficient in describing variations in the position of the EIT resonances as a function of |B|. While 70 nT will certainly not compete with conventional atomic magnetometer measurement methods, it is worth noting that the information needed for this measurement is included for free in the same principal components that can be used for the angular measurement of $\vec{B}$. Furthermore, this magnetometer was not configured to optimize long-term scalar measurement stability, primarily due to environmental fluctuations in the laboratory environment. The observed 70 nT stability may simply reflect the stability of the magnetometer rather than the stability of the PCA post-processing. Regardless, this finding asserts the value in using PCA for vector measurements of $\vec{B}$, given its ability to simultaneously perform magnitude and directionality measurements with EIT spectra.

It is known that magnetometers of this type are capable of achieving pT sensitivity [45]. With phase sensitive detection of the frequency separation of the EIT resonances, we achieved sensitivity better than $10pT/\sqrt{Hz}$ in the 1 Hz–1 kHz frequency range. Preliminary theoretical analysis shows that this result can be improved by an order of magnitude via optimization of the interrogation scheme. The results of this study will be published elsewhere.

## 5. Discussion

The proposed machine learning approach for vector magnetometry does not provide very high accuracy and sensitivity for magnetic field values, but rather is intended for practical use over a large dynamic range of measurements. It is feasible that higher sensitivity could be achieved by incorporating a multistage algorithm in which a coarse prediction of θ is made and then the spectrum is passed on to an assortment of regression models specially optimized for specific ranges of θ. The development of such an algorithm may be the subject of follow-on work in the future development of this technology.

Furthermore, the reported results involve data sets collected over comparably long periods of time with a tabletop breadboard prototype that has not been optimized for low-noise performance. As such, the system is contaminated with many classical noise sources and intrinsic drifts that contribute to a sensitivity reduction of the proposed technique. While multiple data sets were collected over the course of the study, modifications were made to the test setup between the collection of data sets (replacing optical components, changing vapor cells, etc). Because of these changes to the test setup, the principal axes of the EIT spectra are slightly different between the different data sets. The KPCA-SVR model trained with one data set can be used to make accurate predictions for magnetic field values in another data set, but because of the adjustments to the system, there is a small degradation in performance (±2.5°). We believe that this degradation does not reflect a limitation in the proposed methodology, but is simply an artifact from the testbed adjustments. When implementing this protocol in an operational magnetometer, the principal axes of the system should be calculated when other experimental parameters have been optimized and fixed (laser power, RF power, cell temperature, etc).

Despite these limitations, the results indicate that machine learning methods such as the proposed KPCA-SVR techniqe provide a viable alternative to conventional AMO measurement techniques. In what follows, we consider how this technique compares to the conventional methods in spectroscopy, and how the technique might be extended for simultaneous detection of θ and ϕ.

### 5.1. Comparison with conventional methods

To measure the direction of the magnetic field in a more conventional manner, a sensor must identify the relative height of the observed EIT resonances and compare the measurement results with the theoretical predictions [23]. The observation accuracy is hindered by a few technical limitations resulting from a misalignment of the optical beams, inhomogeneity of the magnetic field, deformation of the resonance curves as the result of a buffer gas presence, residual elliptical polarization of the beams, and other reasons. The technical factors leading to asymmetry of the optical spectrum make accurate theoretical evaluation of the field direction difficult.

**Figure 14.**
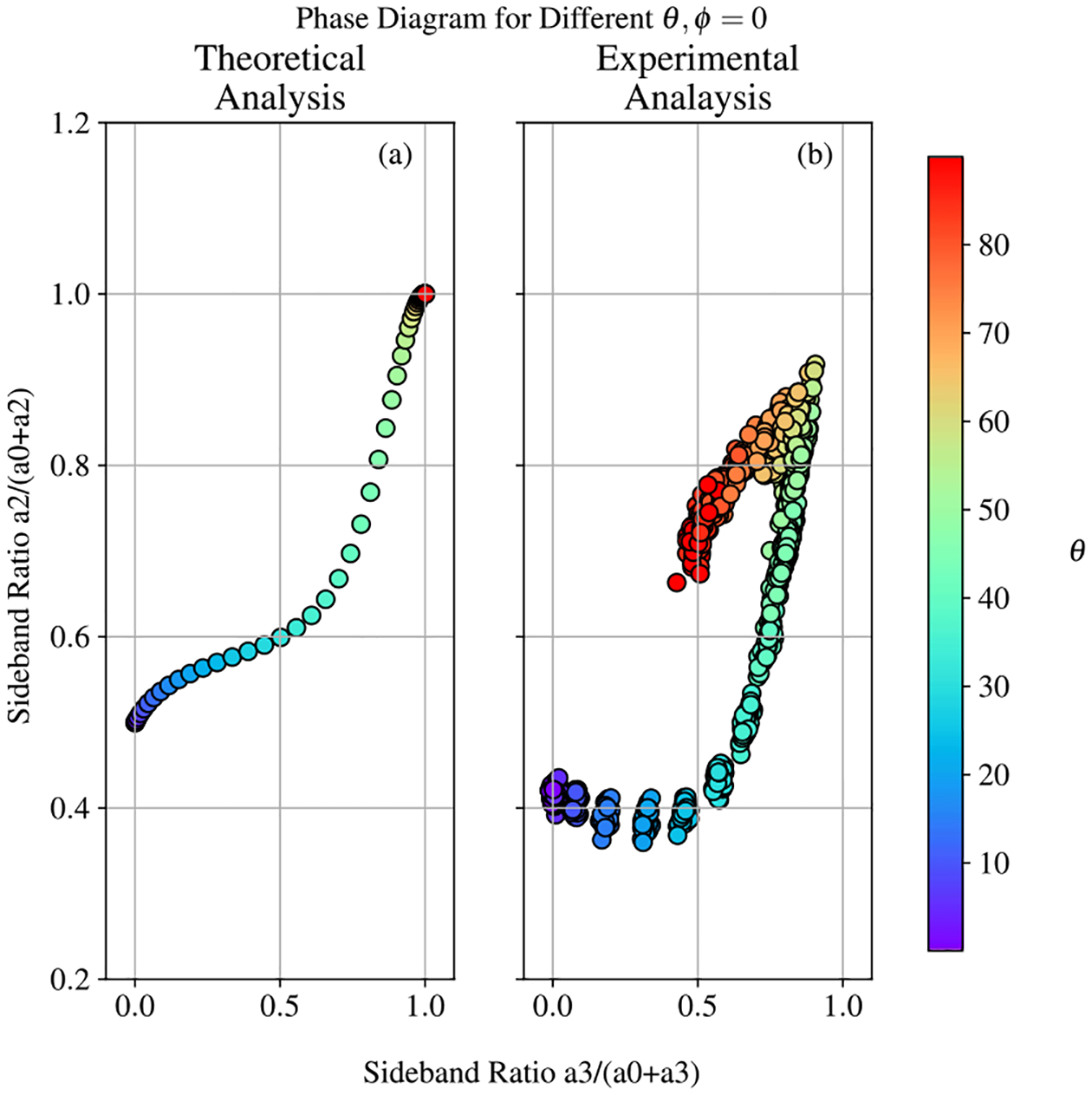
Theoretical numerical, (a), and experimental, (b), distribution of the heights of the EIT harmonics for ϕ=0°. While theory predicts that the measurement allows accurate identification of the angle θ, the experimental data demonstrates reliable results for only a limited range of the angle values.

This statement is illustrated by figure 14, in which the dependence of the spectral peak heights is found both theoretically and experimentally for the case of fixed azimuthal angle ϕ=0. For this analysis, the height of each EIT resonance is measured relative to the background of the measurement, and side band ratios are calculated using the $a_{0},a_{2}$, and $a_{3}$ resonances. In figure 14, the vertical feature is calculated with $a_{2}/\left(a_{0}+a_{2}\right)$ and the the horizontal feature is calculated with $a_{3}/\left(a_{0}+a_{3}\right)$.

**Figure 15.**
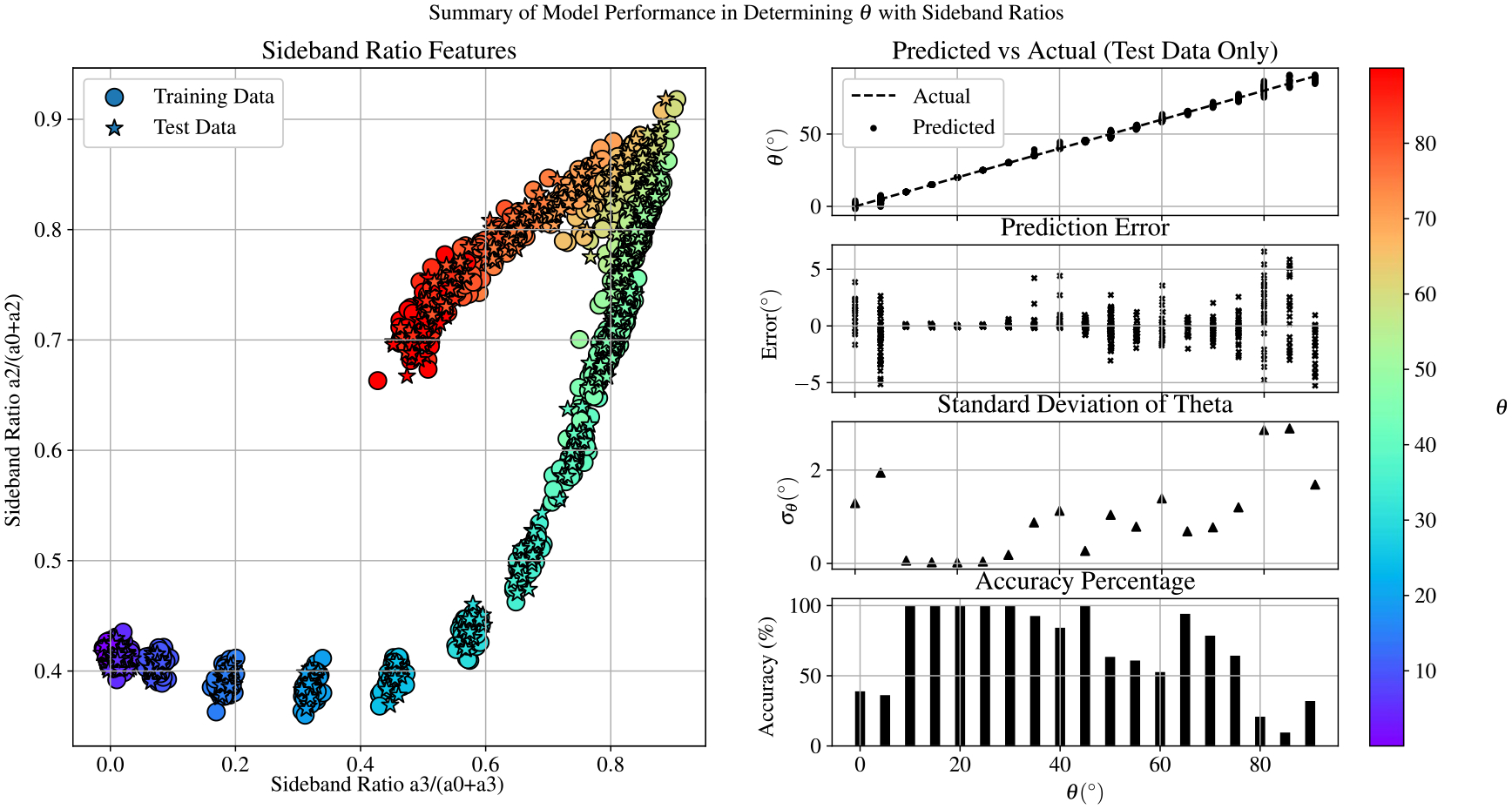
Summary of performance for the sideband ratio-SVR model in recovering θ from EIT spectra. These results illustrate the difficulty in using the conventional AMO measurement techniques to reliably measure the directionality of B for the entire range of θ.

While these curves do not coincide, they do produce features of the same magnitude, and it it is clear that these features possess value in recovering θ. In order to evaluate the predictive power of this method, the side band ratios were also used to train an SVR model using the same procedure and data set outline in section 4(A). The summary for the performance of this method is shown in figure 15. While this model is quite competitive for the 0°<θ<30° range (as expected with the clustering observed in figure 14(b), it is clear that this method fails to accurately predict θ within one degree for most configurations of $\vec{B}$.

**Figure 16.**
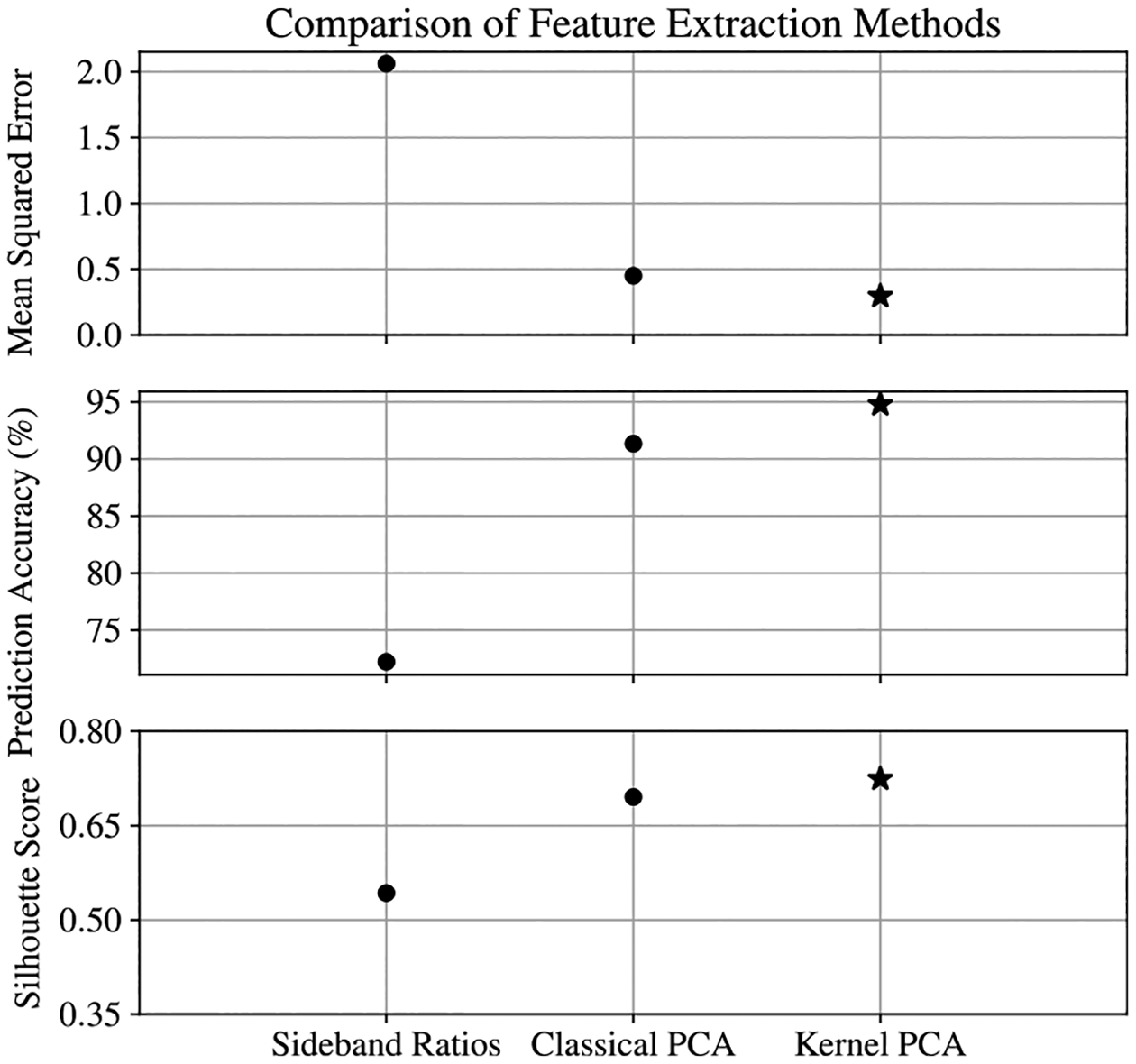
Comparison of the mean squared error, prediction accuracy, and the silhouette score for the three EIT feature extraction methods discussed.

Machine learning methods, like the PCA techniques outlined in this paper, reduce the effect of these systematic errors because they do not rely on a theoretical model of the setup. Any technical imperfections of the system are inherently taken into account when developing data-driven models. By comparing figure 15 against figure 9 and 11, it is clear that the PCA methods outperform the theoretical approach and make predictions for the entire range of directions $\left(0{}^{\circ}<\theta <90{}^{\circ}\right)$ feasible. A performance summary for all three EIT feature extraction methods outlined in this paper is provided in figure 16. Here we see that for all of the figures of merit considered, the PCA methods surpass the theoretical method, and KPCA offers a slight improvement over the classical PCA method. It is worth noting that the time complexity associated with identifying the extrema of each spectrum is approximately O(pn), which is certainly more efficient than any of the PCA techniques. However, the sensitivity to variations in the background and aforementioned technical limitations make this approach less practical for use in uncontrolled environments.

### 5.2. Simultaneous measurement of longitudinal and azimuthal angles

This work has focused on the utility of KPCA in determining only the longitudinal angle, θ, from EIT spectra collected with the magnetometer. Though slightly more challenging, it is possible to extend the methodology to determine the azimuthal angle, ϕ, as well. The measurement is not straight forward and involves observing multiple manifestations of the EIT spectra. In figure 6, we observe that the principal components of the EIT spectra all fall along a smooth curve in the new subspace. However, not only do the projections align on a single curve when ϕ=0°, but the they fall along a single curve for all values of ϕ. This relationship indicates that single manifestations of the EIT spectrum are not unique. As such, if ϕ and θ are unknown, it is impossible to determine the orientation of the magnetic field with only a single trace of the spectrum.

In order to accurately determine θ and ϕ simultaneously, the measurement protocol requires rotating the polarization and observing multiple spectra across variations in ϕ. As ϕ approaches 0°, rotations in the polarization will create significant variations in the EIT spectrum. Conversely, as ϕ approaches 90°, rotations in the polarization will cause minimal variations in the EIT spectrum. Changes in the amount of variation can be quantified with fluctuations in the principal components. This can be seen visually with figure 6, as the separation between clusters for θ=0° and θ=90° decreases as ϕ increases from ϕ=0° to ϕ=90°. The ‘amplitude’ of the spectrum variation can be used to determine the approximate value of ϕ. When ϕ is known, a look-up table or similar tool can be used to determine the value of θ corresponding to the measured spectrum and known ϕ.

While controlling the polarization of the laser and observing multiple spectra is feasible, it increases the time duration of the measurement, and intrinsic drifts within the measurement apparatus begin to degrade the angular sensitivity. With initial tests incorporating this extended measurement approach, we found that the sensitivity of the technique became an order of magnitude worse, resulting in an accuracy on the order of ±5° for recovering ϕ and θ from multiple EIT spectra. In practice, it is more effective to simply rotate the polarization such that ϕ is locked to 0°, measure a single trace of the EIT spectrum, and predict the associated value of θ from the principal components of the measured trace. For this reason, this work has focused only on recovering θ from measurements of the EIT spectrum.

## 6. Conclusion

This paper explored the utility of classical and nonlinear PCA techniques within the context of AMO spectroscopy. PCA provides a means of exploring internal patterns within a collection of data without requiring any assumptions on the underlying physics involved. For the case of optical vector atomic magnetometry, PCA was used to identify the optimal testing configuration for the determination of the angle of the local magnetic field, and KPCA was utilized to train an SVR machine to correlate EIT spectra with specific angles. The demonstrated measurement accuracy was better than one degree for θ if ϕ=0°, and better than three degrees if ϕ and θ are unknown. PCA primarily models the change in the contrast of the EIT resonances for different angles, but further sensitivity improvement is expected if additional fitting parameters are introduced to account for changes in the absolute field. The clustering behavior observed with this EIT magnetometer data set demonstrates that PCA techniques have great potential for addressing regression problems in AMO spectroscopy. Future work may consider a more rigorous uncertainty quantification for angle predictions, or an active shape model that considers only small portions of each spectrum when performing PCA.

## Data Availability

The data cannot be made publicly available upon publication due to legal restrictions preventing unrestricted public distribution. The data that support the findings of this study are available upon reasonable request from the authors.
